# Molecular Dynamics of Functional Azide-Containing Acrylic Films

**DOI:** 10.3390/polym10080859

**Published:** 2018-08-02

**Authors:** Marta Carsí, Maria J. Sanchis, Saul Vallejos, Félix C. García, José Miguel García

**Affiliations:** 1 Instituto de Automática e Informática Industrial, Departamento de Termodinámica Aplicada, Universitat Politècnica de Valencia, 46022 Valencia, Spain; mcarsi@ter.upv.es; 2 Instituto Tecnológico de la Energía, Departamento de Termodinámica Aplicada, Universitat Politècnica de València, Camí de Vera s/n, 46022 Valencia, Spain; 3 Universidad de Burgos, Plaza Misael Bañuelos s/n, 09001 Burgos, Spain; svallejos@ubu.es (S.V.); fegarcia@ubu.es (F.C.G.)

**Keywords:** azide containing polymers, dielectric relaxation spectroscopy, dipolar and conductive processes

## Abstract

A report on the syntheses, thermal, mechanical and dielectric characterizations of two novel polymeric acrylic materials with azide groups in their pendant structures is presented. Having the same general structure, these polymers differ in length of oxyethylene units in the pendant chain [-CONH-CH_2_CH_2_-(O-CH_2_CH_2_)_n_N_3_], where n is 1 (poly(*N*-(2-(2-azidoethoxy)ethyl)methacrylamide), PAzMa1) or 2 (poly(*N*-2-(2-(2-azidoethoxy)ethoxy)ethyl)methacrylamide), PAzMa2), leading with changes in their dynamics. As the thermal decomposition of the azide group is observed above 100 °C, dielectric analysis was carried out in the temperature range of −120 °C to 100 °C. Dielectric spectra of both polymers exhibit in the glassy state two relaxations labelled in increasing order of temperature as *γ*- and *β*-processes, respectively. At high temperatures and low frequencies, the spectra are dominated by ohmic conductivity and interfacial polarization effects. Both, dipolar and conductive processes were characterized by using different models. Comparison of the dielectric activity obtained for PAzMa1 and PAzMa2 with those reported for crosslinked poly(2-ethoxyethylmethacrylate) (CEOEMA) was performed. The analysis of the length of oxyethylene pendant chain and the effect of the methacrylate or methacrylamide nature on the dynamic mobility was analysed.

## 1. Introduction

Reactive and functional organic polymers typically consist of regular polymers with specific chemical groups attached to the backbone that provide them with specific chemical reactivity, or behaviour. This can, in turn, be interesting for application purposes where such polymers can be useful to facilitate specific chemical reactions to obtain semi conductive, stimuli-responsive, supramolecular, cross-linkable, catalyst, or biomimetic polymers. From a practical point of view, such systems are very interesting because the appropriate attaching of selected chemical groups via synthetic techniques may offer a viable way to modify and tune the physical and/or chemical properties of polymers. However, to take full advantage of the potential of these systems, the general effects of functionalization on the polymer dynamics and subsequent changes on polymer properties, must be understood.

Among the many functional groups available, the organic azide group is especially interesting because it can be considered to be a building block [1,2]. In the field of radical polymerization, azide group is probably one of the most studied because of the great possibilities that their relatively easy synthesis offers. Additionally, this group can be transformed into several organic groups and structures by reaction with both electrophiles and nucleophiles [1,2]. Thus, for example, the azide group is used in click reactions, such as the widely exploited azide-alkyne cycloadditions [3,4,5]. Furthermore, the nitrene group can easily be generated from the azide group, both thermally and photo chemically and used without isolation in cycloadditions, C-N insertions and ring-expansions, to name but a few. Additionally, it is usually thermally stable below 100 °C [1]. In this sense, it allows the easy polymerization of a varied set of sophisticated sensitive active polymers. In order to improve our knowledge of the potential uses of azide containing polymers for advanced applications, we are currently working on the derivatization of the polymers described in this work to prepare ferrocene electro active sensory materials, polymers with amino groups for enzyme immobilizations and polymers with pendant biomolecule motifs, such as dopamine. Additionally, the organic azide group presents a strong dipole moment that, from a fundamental point of view, can be useful for selectively studying specific polymer dynamics using broadband dielectric spectroscopy (BDS).

According to the special interest that azide-containing organic polymers arouse, we prepared two novel polymeric acrylic materials with azide groups in their pendant structures in well-defined and predetermined positions along the chain. The two polymers differ in length of oxyethylene pendant chain [-CONH-CH_2_CH_2_-(O-CH_2_CH_2_)_n_N_3_], where n is 1 or 2. The molecular dynamics of polymers are highly sensitive to the composition and polymer chain architecture. In regard to any commercial application for acrylic polymer films, molecular dynamics are of special interest as a consequence of the direct relationship existing between structure and properties and consequently, potential use. Therefore, this type of analysis is really interesting for the identification and tailoring of materials suitable for specific applications.

Herein, we present the first results from a systematic study of the dynamics of two novel polymeric acrylic materials having pendant structures with azide groups by BDS as this is one of the most powerful methods to study dynamic relaxation properties. The mean advantage of DRS from dynamic mechanical analysis (DMA) technique lies in their possibility of analysis of the chain’s response over more than 10 decades in the frequency domain [6,7,8]. However, a disadvantage of the BDS technique is that in sometimes, it is not possible the analysis of the relaxation process related to the glass transition due to the masking of this process by the dominant conductive processes [7,8]. In an earlier work, the dielectric and mechanical relaxations of lineal poly(2-ethoxyethyl methacrylate) (PEOEMA) and crosslinked poly(2-ethoxyethyl methacrylate) (CEOEMA) has been investigated and it has been found that both show a variety of absorptions due to the versatility of their structural moiety [9,10]. The last polymer is structurally related to poly *N*-(2-(2-azidoethoxy)ethyl)methacrylamide (PAzMa1) sample, that is, both acrylic polymers with pendant flexible oxyethylene units, where a methacrylate group is changed by a methacrylamide group and the terminal side chain hydrogen is replaced by an azide unit.

The aim of this paper is to study the effect of a slight modification in the chemical structure of the two methacrylamides ([R-CONH-CH_2_CH_2_-(O-CH_2_CH_2_)_n_N_3_], n = 1 or 2) on the molecular mobility of polymers containing azides groups in the side chains and their comparison with other related methacrylate polymers. DRS was used to analyse the dipolar and conductive processes. An analysis of both dipolar and conductivity processes has been carried out.

## 2. Materials and Methods 

### 2.1. Materials

All materials and solvents were commercially available and used as received, unless otherwise indicated: Triethylene glycol (Aldrich, Madrid, Spain, >99%), diethylene glycol (Aldrich, 99.5%), p-toluenesulfonyl chloride (Aldrich, >98%), potassium hydroxide (VWR, Radnor, Pennsilvània, Estats Units, >85%), dichloromethane (Acros Organics, Morris, NJ, USA, 99.9%), sodium sulphate (Aldrich, 99.99%), sodium azide (Merck, Darmstadt, Germany, 99.5%), tetrabutylammonium iodide (Aldrich, 98%), dimethylformamide (Aldrich, 99.8%), diethyl ether (Aldrich, >99.7%), tetrahydrofuran (Aldrich, >99.9%), hydrochloric acid (VWR, 36.5%), triphenylphosphine (Aldrich, 99%), methacrylic anhydride (Aldrich, 94%), ethyl acetate (VWR, 99.8%), ethyleneglycoldimethacrylate (Alfa Aesar, Haverhill, Massachusetts, Estats Units, 98%), azobisisobutyronitrile (Aldrich, 98%).

The synthesis of *N*-(2-(2-azidoethoxy)ethyl)methacrylamide, 2-(2-azidoethoxy)ethanamine and 2-(2-(2-azidoethoxy)ethoxy)ethanamine was carried out starting from diethylene glycol and triethylene glycol following a previously described procedure [11].

### 2.2. Monomer Synthesis

*N*-(2-(2-azidoethoxy)ethyl)methacrylamide (AzMa1): Methacrylic anhydride (2.15 g, 13.96 mmol) and 2-(2-azidoethoxy)ethanamine (1.65 g, 12.69 mmol) were dissolved in 60 mL of ethyl acetate in a pressure round bottom flask. The solution was stirred at 100 °C for 4 h. Then, the reaction was cooled to room temperature (rt) and washed with an aqueous solution of sodium hydroxide. Finally, the organic layer was dried over sodium sulphate and the solvent was removed under vacuum. The liquid product was purified by the following procedure: (a) mixing with the minimum quantity of hot heptane to form a unique phase; (b) filtering off; (c) cooling with an ice-bath; (d) after cooling, two phases were obtained and the solvent eliminated by decantation.

*N*-(2-(2-(2-azidoethoxy)ethoxy)ethyl)methacrylamide (AzMa2) was prepared and purified following the procedure followed to prepare AzMa1 starting from methacrylic anhydride (170 mg, 1.1 mmol) and 2-(2-(2-azidoethoxy)ethoxy)ethanamine (175 mg, 1 mmol).

### 2.3. Sample Preparation

**Figure 1:**
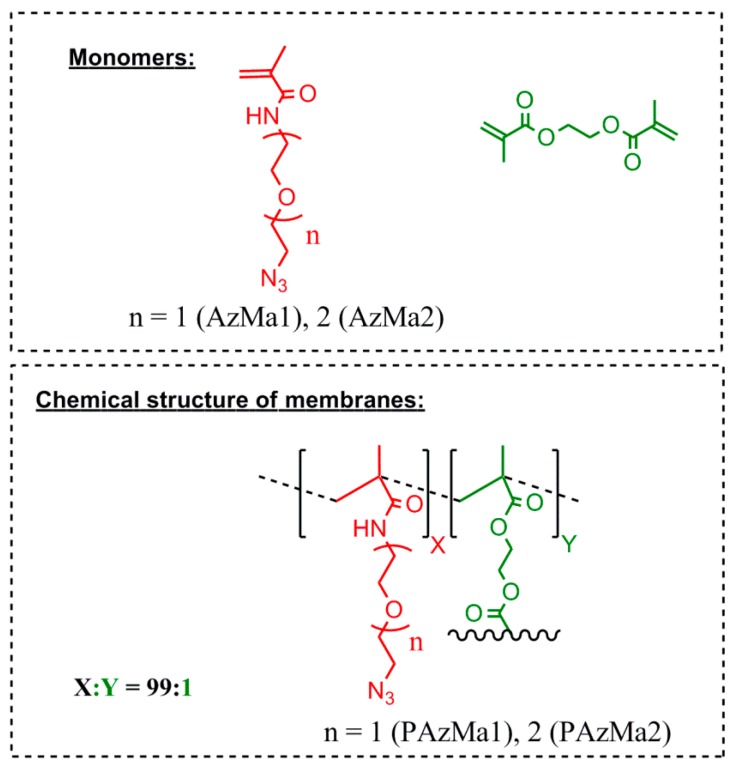
Chemical structure of films, PAzMa1 and PAzMa2 and monomers used in their preparation.

Both films (PAzMa1 and PAzMa2) were prepared by radical polymerization of the respective monomers (AzMa1 and AzMa2), using a small molar amount (1%) of ethyleneglycoldimethacrylate (EGDMA) as a crosslinking agent and AIBN (1 wt %) as thermal radical initiator. The bulk radical polymerization reaction was carried out in a silanized glass mould that was 100 μm thick in an oxygen-free atmosphere at 60 °C for 20 h. Then, the films were demoulded and washed in acetone to remove non-reacted monomers and other impurities. Finally, before their characterization, the films were washed with distilled water and dried in air at rt. The chemical structure of monomers and films is depicted in Figure 1.

### 2.4. Nuclear Magnetic Resonance Spectroscopy (NMR)

^1^H and ^13^C NMR spectra were recorded with a Varian Unity Inova (Varian, Palo Alto, CA, USA) 400 spectrometer operating at 399.94 and 100.58 MHz, respectively.

### 2.5. Fourier Transform Infrared Spectroscopy (FTIR)

Infrared spectra (FTIR) of dried PAzMa1 and PAzMa2 samples were recorded with a JASCO FT/IT-4100 (Tokyo, Japan) fitted with a PIKE TECH “MIRacle” ATR (Madison, WI, USA) accessory.

### 2.6. Thermal Gravimetric Analysis (TGA)

Thermogravimetric analysis (TGA) data of PAzMa1 and PAzMa2 were obtained for dried samples of 5 mg under nitrogen flow, using a TA Instruments Q50 TGA analyser (New Castle, DE, USA) at a scan rate of 1 °C·min^−1^.

### 2.7. Differential Scanning Calorimetry Analysis (DSC)

DSC measurements of PAzMa1 and PAzMa2 dried samples were carried out using a TA instruments Q200 DSC (New Castle, DE, USA) with a refrigerated cooling system. Thin films were repeatedly stacked into a pan, with a weight of 10 mg. The measurements were conducted in crimpled non-hermetic aluminium pans, using an empty pan as reference. The DSC tests were performed under a 50 mL·min^−1^ flow of nitrogen to prevent oxidation. High-purity indium was used to calibrate the DSC cell. Thermograms were obtained in the heating ramp from 0 to 180 °C at a scan rate of 40 °C·min^−1^. *T_g_* was evaluated as the intersection of the baseline of the glassy region with the tangent to the endothermal the middle points. High heating scan rate allowed for both the detection of the transition and for minimizing the exposure of the material to high temperatures, where the decomposition of the azide groups takes place but at the expense of less the temperature resolution and accuracy.

### 2.8. Mechanical Properties

The tensile properties of PAzMa1 and PAzMa2 were analysed using a Shimadzu EZ Test Compact Table-Top Universal Tester (Kyoto, Japan). Strips were cut from the polymer films with 5 mm of width, 30 mm of length and 100−120 μm of thick. The samples were dried at 60 °C for 1 h and then immediately measured with an extension rate of 5 or 10 mm·min^−1^ and a gauge length of 10 mm. At least six samples were tested for each polymer and the data were averaged.

### 2.9. Dielectric Relaxation Spectroscopy (DRS)

Isothermal relaxation spectra of PAzMa1 and PAzMa2 samples were collected by using a Novocontrol Broadband Dielectric Spectrometer (Hundsagen, Germany) consisting of an Alpha analyser to carry out measurements from 5 × 10^−2^ to 3 × 10^6^ Hz. The measurements were performed in an inert N_2_ atmosphere from −120 °C to 100 °C. The temperature was controlled by a nitrogen jet (QUATRO from Novocontrol, Montabaur, Germany) with a temperature error of 0.1 °C during every single sweep in frequency. Moulded disc shaped samples of about 0.1 mm thickness and 20 mm diameter were used. In order to avoid the increase of conductivity due to the water, before being inserted into the instrument, the samples were placed under vacuum until constant weight at 40 °C. The experimental uncertainty was better than 5% in all cases.

## 3. Results

### 3.1. Synthesis of the Monomers and Preparation of the Films 

**Figure 2:**
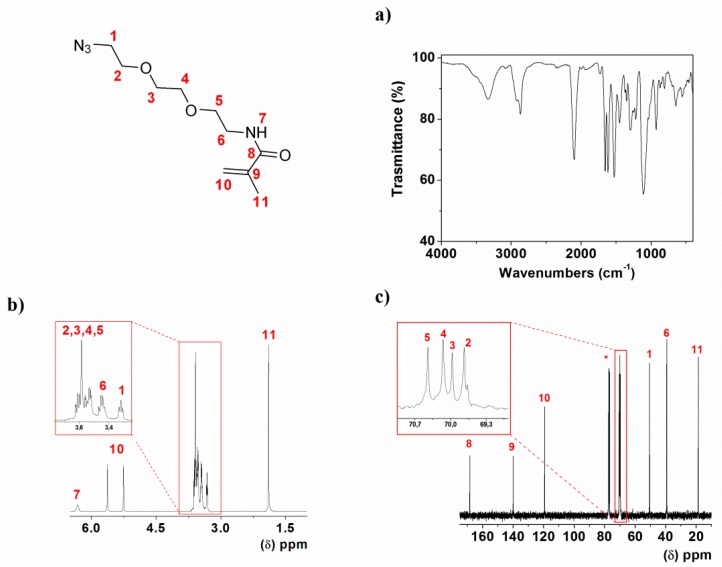
Characterization of AzMa2 by (**a**) FTIR, (**b**) ^1^H-NMR and (**c**) ^13^C-NMR (* = solvent signal, CDCl_3_).

The monomers, AzMa1 and AzMa2, were straightforwardly prepared from inexpensive and widely available materials starting with di and triethyleneglycol, respectively. Three previously described synthetic steps rendered 2-(2-(2-azidoethoxy)ethoxy)ethanamine and 2-(2-azidoethoxy)ethanamine from the corresponding glycol with an overall yield of 55% [12]. The reaction of these compounds with methacrylic anhydride rendered the liquid monomers with high yield, which were easily purified by treating them with hexane to give high purity chemicals, as shown by FTIR, ^1^H and ^13^C NMR for AzMa2 (Figure 2). *N*-(2-(2-azidoethoxy)ethyl) methacrylamide (AzMa1): Yield 70%. ^1^H-NMR (399.94 MHz; DCCl_3_): δ (ppm), 6.28 (s, 1H); 5.66 (s, 1H); 5.27 (s, 1H); 3.62 (m, 2H); 3.54 (m, 2H); 3.48 (m, 3H); 3.32 (t, 2H, 4.82 Hz); 1.90 (s, 3H). ^13^C-NMR (100.58 MHz; DCCl_3_): δ (ppm), 168.37, 139.73, 119.67, 70.02, 69.75, 50.56, 39.29, 18.52. EI-HRMS, (M + H)^+^: 199.1186. FT-IR (Wavenumbers, cm^−1^): ν_NH_: 3329; ν_as,N3_: 2099; ν_C=O_, amide I: 1658; ν_as,C-O-O_ = 1119. *N*-(2-(2-(2-azidoethoxy)ethoxy)ethyl)methacrylamide (AzMa2): Yield 85%. 1H-NMR (399.94 MHz; DCCl3): δ (ppm), 6.31 (s, 1H); 5.63 (s, 1H); 5.26 (s, 1H); 3.58 (m, 8H); 3.44 (m, 2H); 3.32 (t, 2H, 4.4Hz); 1.89 (s, 3H). ^13^C-NMR (100.58 MHz; DCCl_3_): δ (ppm), 168.39, 139.96, 119.38, 70.45, 70.15, 69.97, 69.73, 50.54, 39.27, 18.57. EI-HRMS, (M + H)^+^: 243.1449. FT-IR (Wavenumbers, cm^−1^): ν_NH_: 3329; ν_as,N3_: 2099; ν_C=O_, amide I: 1657; ν_as,C-O-O_ = 1113.

Films preparation was accomplished following conventional bulk thermally initiated radical polymerization. The monomers containing the azide functionality AzMa1 or AzMa2 were polymerized using a small molar amount (1%) of EGDMA as a crosslinking agent and a small amount of thermal radical initiator. The film-shaped films were about 125 μm thick, highly transparent and colourless, creasable and tractable.

### 3.2. Fourier Transform Infrared Spectroscopy (FTIR) 

**Figure 3:**
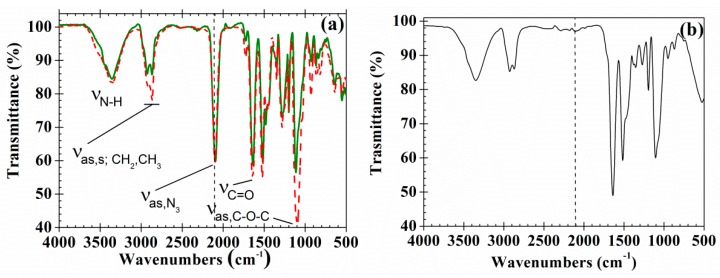
(**a**) Normalized (ν_as,N3_ peak) FTIR spectra of PAzMa1 (continuous line) and PAzMa2 (dash dot line) films and (**b**) FTIR spectra of PAzMa1 after thermal treatment (heated in TGA from rt to 250 °C at 1 °C·min^−1^).

The FTIR spectra of PAzMa1 and PAzMa2 (Figure 3a) show the typical bands of the azide and amide groups (ν_N-H_: 3355 cm^−1^; ν_as,N3_: 2095 cm^−1^; ν_C=O, amide I_: 1642 cm^−1^), along with the typical bands of the stretching of the pendant ether linkages (ν_as,C-O-C_ = 1095 cm^−1^) and asymmetric and symmetric stretching bands of the methyl and methylene groups (ν_as; CH3,CH2_ = 2921 cm^−1^; ν_s; CH3,CH2_ = 2867 cm^−1^). The bands corresponding to the presence of ether and methylene groups are more intense in PAzMa2 than in PAzMa1 due to the longer side chain in the former.

The thermal treatment of the films (from rt to 250 °C at 1 °C·min^−1^) leads to crosslinked materials due to the nitrogen evolution with the formation of the highly reactive nitrene group, a diradical that rapidly undergoes further reactions, as described below. The disappearance of the azide band at ca. 2095 cm^−1^ can be used to follow this reaction (Figure 3b).

### 3.3. Thermal Gravimetric Analysis Characterization (TGA) 

**Figure 4:**
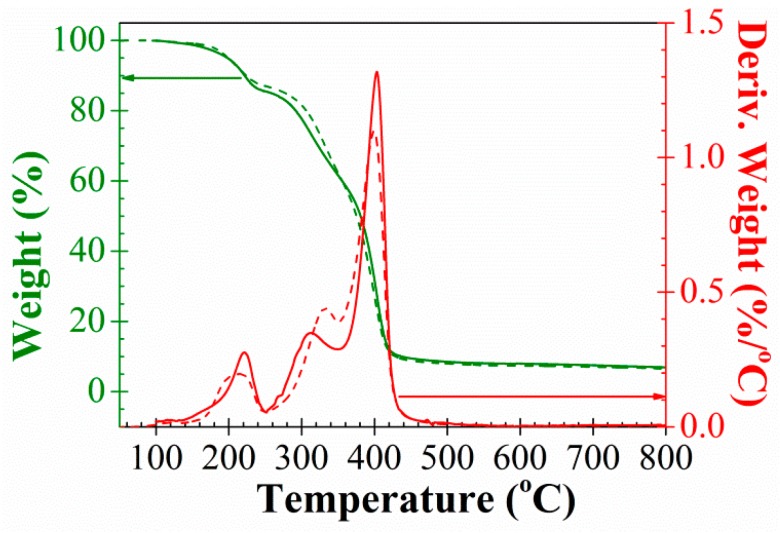
TGA and first derivative TGA curves of PAzMa1 (continuous line) and PAzMa2 (short dot line) samples.

Thermal stability of materials was analysed by TGA. Figure 4 shows the TGA spectra obtained for PAzMa1 and PAzMa2. The weight loss of 5 and 10% is observed for both films at about 200 °C and 220 °C (Table 1). However, the thermal decomposition of the azide group is observed from 100 °C, showing maxima decomposition speed at about 220 °C and the end of the decomposition temperature at 240 °C. The decomposition of the azide group gives rise to the formation of nitrogen and a highly reactive nitrene, a diradical species, which rapidly results in cross-linking reactions by self-coupling to render azo groups and other reactions with the functional groups present in the material [12,13,14,15]. The weight loss at 240 °C corresponds to the loss of one molecule of nitrogen per azide group. Immediately after the loss of nitrogen, the degradation pattern of the films is similar to that of dry polyacrylamide, where the breakage of the amide group, with the liberation of ammonia and formation of imide groups, initiates as 230 °C and have a maximum degradation rate at 313 °C [16]. This breakage generates in our films low molecular weight fragments with degradation rate maxima observed at lower temperatures for the polymer with shorter side chains (311 °C for PAzMa2 and 334 °C for PAzMa2).

### 3.4. Differential Scanning Calorimetry Characterization (DSC)

**Figure 5:**
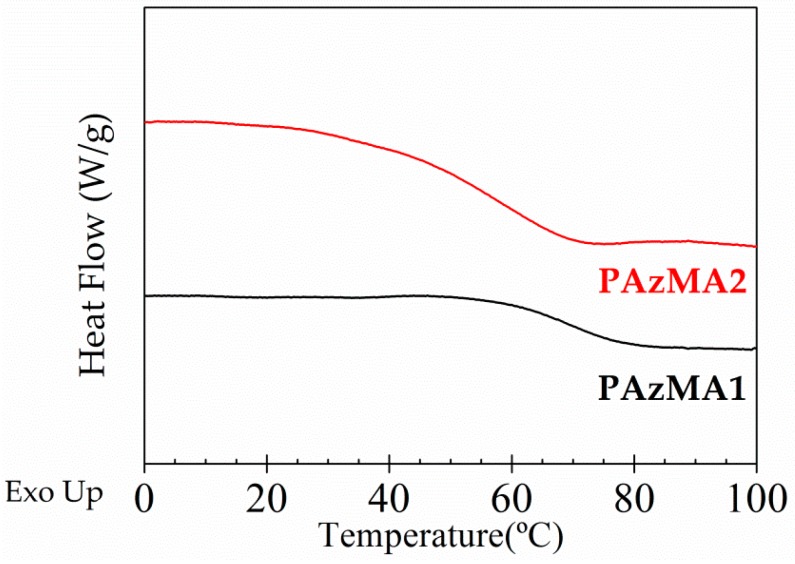
DSC experimental curves for PAzMa1 (bottom) and PAzMa2 (above) samples.

The DSC thermograms obtained for both samples, plotted in Figure 5, exhibit well developed endotherms associated with the glass transition temperature. The obtained *T_g_* values, estimated as the temperature at the midpoint of the endotherms, were 67 °C and 57 °C for PAzMA1 and PAzMa2 samples, respectively. Thus, the increase of the length of the side chain increases the chain mobility with the consequent reduction of *T_g_* (about 10 °C).

### 3.5. Mechanical Properties

The films were mechanically flexible and creasable, having Young’s modulus ranging 246–778 MPa, tensile strength between 12 and 25 MPa and deformation at break between 4 and 14. According to the results collected in Table 1, increasing the length of the side chain, lower Young’s modulus and tensile strength and higher deformation at break were obtained. Thus, increasing the length of the side chain, the flexibility rises.

### 3.6. Dielectric Relaxation Spectroscopy (DRS)

**Figure 6:**
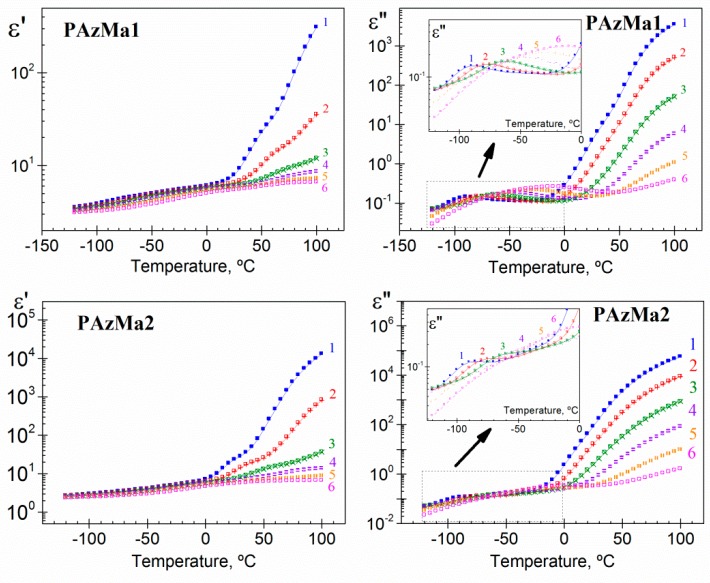
Real and loss components of the complex dielectric permittivity as a function of temperature for PAzMa1 and PAzMa2 at 1.19 × 10^0^ [1: square], 8.73 × 10^0^ [2: ballot box with +], 9.52 × 10^1^ [3: ballot box with ×], 1.04 × 10^3^ [4: square with top half black], 1.13 × 10^4^ [5: square with right half black] and 1.2 × 10^5^ [6: square containing black central point].

Isochrones, at several frequencies, showing the variation of the real (*ε’*) and imaginary (*ε”*) components of the dielectric complex permittivity (ε*) with temperature for both polymers, are shown in Figure 6. In order of increasing temperature, the *ε’* isochrones slightly increase with temperature in the glassy state and undergo a steep increase at high temperatures due to the vicinity of the glass transition temperature and of the presence of conductive processes. On the other hand, with increasing temperature, the loss isochrones of the dielectric loss, *ε”*, exhibit two weak relaxations, named *γ* and *β* (in the glassy state), followed by a continuous increase associated with the overlapping of the glass-liquid or *α*-relaxation and the conduction phenomena, which includes both ohmic and non-ohmic conduction. The latter can take place at inner dielectric boundary layers on a mesoscopic scale, Maxwell-Wagner-Sillars polarization (MWS) and/or at the external electrodes contacting the sample on a macroscopic scale, electrode polarization (EP) [7,8]. The last contribution is related to the accumulation of charges at the electrode-polymer interface, mainly related to sample impurities. That is, this process is related to the concentration of free ions and does not represent an intrinsic characteristic of the materials investigated. As a consequence of the strong conductivity contribution to the loss permittivity, mainly at higher temperatures and lower frequencies, the *α*-relaxation is hardly discernible in the dielectric loss spectra.

Several different approaches have been used in order to reveal the existence of small intensity processes compared to the conductive signal, in the dielectric loss spectrum [17,18,19,20,21]. One of them, is based on the use of the alternative representation, in terms of the dielectric modulus, which can be evaluated from the permittivity as M*(*ω*) = 1/ε*(*ω*). Unfortunately, this alternative representation did not permit a clear viewing of the *α*-relaxation again due to overlapping of this relaxation process with the conduction phenomena. With the objective to bridge this gap several alternatives have been employed. One of them, is based on the fact that both the real and imaginary parts of the complex contain the same information and both are interrelated by the Kramer-Kroning relations [22]. So, as permittivity, *ε′*, is not affected by dc-conductivity as long as EP remains negligible, this constant can be used to obtain the loss permittivity without pure ohmic conduction contribution. This method, labelled as Wübbenhorst analysis [17,23], is a derivative method of analysis based on the Kramer-Kroning relation. It is usually presented as, ${\varepsilon^{\prime \prime }}_{der}=-\left(\pi /2\right)\cdot \left(d\varepsilon^{\prime }/d\ln\omega \right)$, where *ω* (=2*πf*) is the angular frequency of the electric field.

**Figure 7:**
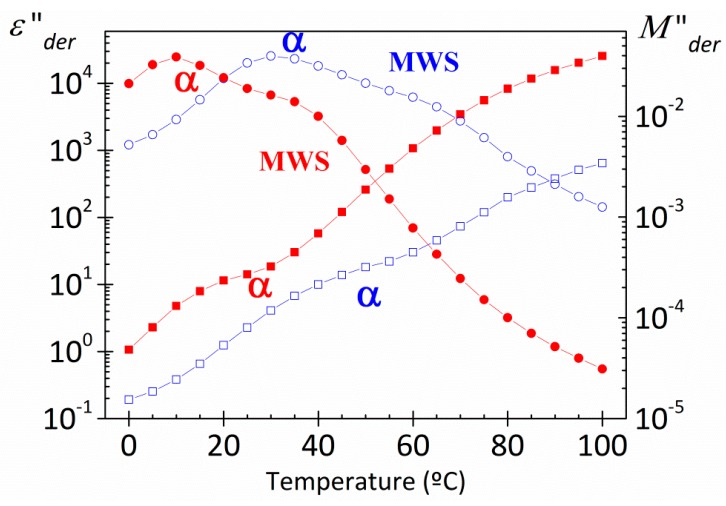
Temperature dependence of the loss permittivity (square) and loss modulus (circle) derivative spectra of the PAzMA1 (open symbols) and PAzMA2 (full symbols) at 1.19 × 10^0^ Hz.

Figure 7 shows, as an example, the loss dielectric spectra obtained from this derivative method for both PAzMA samples at 1.19 Hz. Although there is a better definition of *α*-relaxation, it is still not well defined, due to the overlap with another process. In this Figure we have plotted also the dielectric results in terms of the derivate loss modulus, showing themselves the presence of two overlapping processes in the temperature zone of the glass transition temperature evaluated by DSC .It should be noted that the dipolar relaxation peaks (*M”*) appear shifted to higher frequencies and low temperatures with respect to permittivity representation (*ε”*), as would be expected, [(*τ_ε_*/*τ_M_*) = (*ε_0_*/*ε_∞_*), where *ε_0_* and *ε_∞_* denote the unrelaxed and relaxed parts of the real component of the complex dielectric permittivity, respectively]. If we have eliminated the ohmic conductivity, what is the nature of the second process visualized in the spectrum? Based on results recently obtained by us with other materials with structural similarities with the materials analysed in this work [9,10], we can point out that the origin of the high temperature process in the spectrum obtained, is related to the presence of MWS process. That way, the PAzMA1 sample displays some structural similarities with the cross-linked poly(2-ethoxyethyl methacrylate) (CEOEMA) sample analysed previously by us [9,10]. So, the terminal side chain hydrogen has been replaced by an azide unit and a –COO– (methacrylate) is replaced by a –CONH– (methacrylamide). In addition, the chemical nature of cross-linker is the same in both samples have. As reported by us, the dielectric spectrum of uncross-linked poly(2-ethoxyethyl methacrylate) (PEOEMA) show two secondary relaxations (*γ* and *β*), arising from motions of the side chains that change the orientation of the ether and ester dipoles, followed at lower frequencies by a well-defined *α*-relaxation and conductivity processes [9]. With the addition of 2.5% of cross-linker (EGDMA) to PEOEMA, in the dielectric spectrum of the resulting sample (CEOEMA), the *β*-relaxation is masked and a well-defined relaxation attribute to a Maxwell−Wagner−Sillars (MWS) process, arising from the transport of electric charges in segregated nanodomains formed by the side chains surrounded by the skeletal bond, is shown. Probably, the nanodomains presence hinders the movement of molecular chain units responsible for *β*-relaxation [9]. Although the cross-linker content in the PAzMa samples is lower, at first sight, in loss permittivity experimental spectra, it is not possible to observe the existence of the MWS process but *β*-relaxation is visible. Thus, if the nanodomains exist, the movement restriction imposed by them is not significant to prevent the visualization of this dipolar relaxation process. Therefore, according to the dielectric analysis made in CEOEMA sample and the results showed in Figure 7, it is very likely that the processes that appear at high and low temperatures in the derivate loss dielectric spectra (*ε”* and *Μ”*) are associated, respectively, with a MWS process and with the *α* relaxation dielectric process, related to the glass transition temperature.

**Figure 8:**
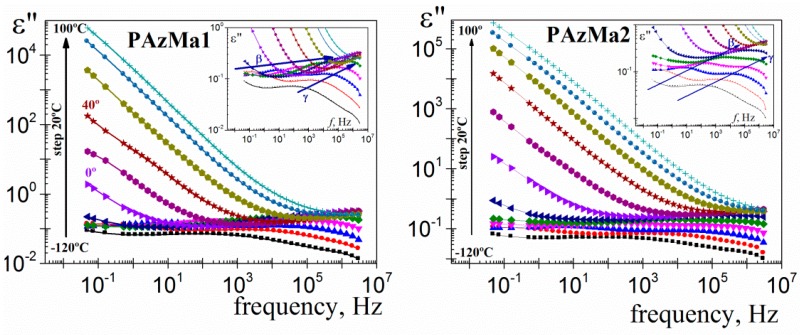
Dielectric loss permittivity as a function of frequency for PAzMa1 and PAzMa2 in the temperature range −120 °C–100 °C (at 20 °C steps).

For the convenience of further analysis and calculations, the dielectric permittivity loss was also plotted in the frequency domain. Thus, Figure 8 shows the isotherms for the dielectric loss for the two PAzMA samples analysed. Both samples, for low temperature isotherms (inset Figure 8), present two dipolar relaxations labelled, in decreasing order of frequency, as *γ* and *β* relaxation processes. These relaxation processes appear at similar temperatures that in previously analogous polymers [9]. Therefore, it is reasonable to postulate that the origin of the observed relaxation is presumably associated with similar motions to those one assigned to CEOEMA. In that case, motions of the side chains that change the orientation of the methacrylamide dipoles for the *γ* relaxation process and local motions of the backbone combined with motions of the side groups for the *β* relaxation process. Finally, at low frequency and high temperature, the spectrum is dominated by strong conductive processes, which, as in the previous representation, hiding the *α*-relaxation, associated with the glass transition.

**Figure 9:**
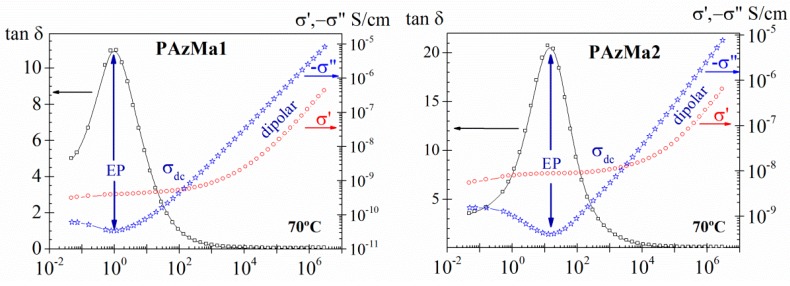
Frequency dependence of tan *δ* and of the real (*σ’*) and imaginary (−*σ”*) parts of the complex conductivity (σ*) for PAzMa1 and PAzMa2 at 70 °C.

For the purpose of visualizing and analysing the conductive contributions to the dielectric permittivity, particularly important at low frequencies, it is also convenient to analyse the results in terms of other dielectric constants, such as tan *δ* and the complex conductivity parameters, which are very sensitive to this type of phenomenon. The complex conductivity can be expressed in terms of dielectric permittivity as $\sigma^{*}\left(\omega \right)=i\omega e_{0}\varepsilon^{*}\left(\omega \right)$, where e_0_ (8.854 pF m^−1^) is the dielectric permittivity of the empty space. As an example, in Figure 9, are plotted, for both samples, the frequency dependence of the tan *δ* and of the complex conductivity for the 70 °C isotherm. In this Figure, we can observe that at low-frequency zone, frequency-independent conductivity (*σ′*) is recorded, which is attributed to resistive conduction through the bulk of the polymer. In contrast, when the frequency is increased, the main displacement of the charge carriers is reduced and the conductivity (*σ′*) becomes proportional to frequency. The observed plateau deviation, at the lower frequencies, in *σ′* representation is related to the EP process, which is visualized, respectively, as a peak and as a minimum in tan *δ* and *σ”* representations.

**Figure 10:**
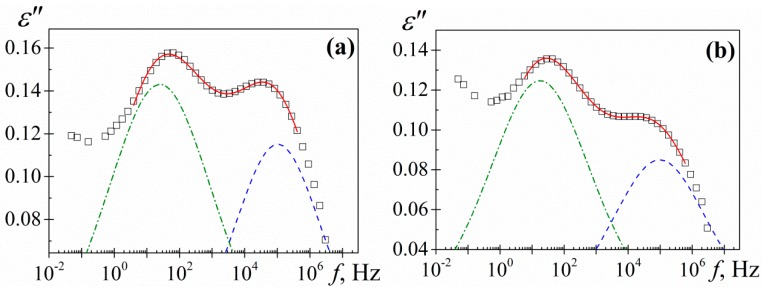
Deconvolution of loss factor for (**a**) PAzMa1 and (**b**) PAzMa2 at −70 °C. Squares represent the experimental data, continuous line the HN fitting curve and dashed lines the individual processes.

The analysis of the overlapping *γ* and *β* processes was carried out, assuming an additive rule [24] for the permittivity, by means the empirical Havriliak-Negami (HN) equation [25,26,27]

(1) $$\varepsilon^{*}\left(\omega \right)-\varepsilon_{\infty }=\sum_{i=\beta ,\gamma }\frac{\varepsilon_{0i}-\varepsilon_{\infty i}}{{\left[1+{\left(i\omega \tau_{i}\right)}^{a_{i}}\right]}^{b_{i}}}\;$$

where the subscript *i* refers to the absorptions *β* and *γ*, while the subscripts 0 and ∞ mean, respectively, relaxed (*ω* = 0) and unrelaxed (*ω* = ∞) dielectric permittivity. The *τ_i_* denotes the relaxation time associated with the *i*-process and the *a_i_* and *b_i_* shape parameters are related, respectively, to the departure of the complex *ε’* versus *ε”* plot from a semi circumference at low frequencies (the lower the value of *a*, the higher departure) and to the skewness of the plot along a straight line, at high frequencies. For secondary processes *b* = 1. The HN parameters of the two secondary relaxations were determined at several temperatures from a multiple nonlinear regression analysis of the *ε”* experimental data, allowing the three characterizing peak parameters (Δ*ε*_i_, *τ*_i_, a_i_) to vary. As an example, the deconvolution of *γ* and *β* processes for the −70 °C isotherm is depicted in Figure 10.

**Figure 11:**
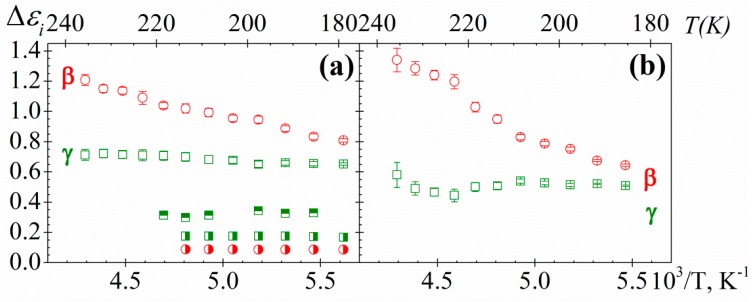
Temperature dependence of the strength for *γ* (squares) and *β* (circles) relaxations of (**a**) PAzMa1 and (**b**) PAzMa2 samples. In (**a**) has been shown the strength relaxation values corresponding to the PEOEMA (square with right half black, circle with right half black) and CEOEMA (square with top half black) samples [9].

The temperature dependence of the relaxation strength (Δ*ε*) for both samples and processes is shown in Figure 11. This dependence follows the classical trends, that is, increases (*β*-relaxation) or nearly remains constant (*γ*-relaxation) with increasing temperature. This tendency is consistent with a thermally activated mechanism, due to the dipole mobility increases with temperature, connected with the free volume increase. Non-significant changes in Δ*ε* for both relaxations with the length of the side chain are observed. However, the Δ*ε* of the secondary relaxations of PAzMa1 is significantly higher than that observed in the structurally related crosslinked polymer, CEOEMA [9]. Considering that Δ*ε* of the secondary relaxations is proportional to (i) the number of entities per volume unit involved in the relaxation process and (ii) the corresponding dipole moment, accordingly the Onsager-Fröhlich-Kirkwood (OFK) equation [28], this significant difference of the Δ*ε*_i_ values for PAzMa1 and CEOEMA must be related to the two structural modifications added. Thus, the incorporation of a terminal azide group and the change of the –COO– group by the –CONH– group into the side chain favour one or both factors. In PAzMa1, we have introduced a polar azide group in the side chain and additionally, the net dipolar moment of the –CONH– is significantly higher than that corresponding to the –COO– [29].

**Figure 12:**
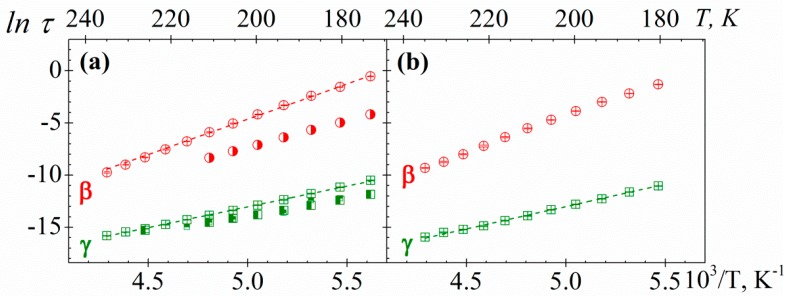
Arrhenius plot for *γ* (squares) and *β* (circles) relaxations of (**a**) PAzMa1 and (**b**) PAzMa2 samples. In (**a**) has been shown the time relaxation values corresponding to the PEOEMA (square with right half black, circle with right half black) and CEOEMA (square with top half black) samples [9].

The temperature dependence of the relaxation times, associated with the peak maxima of the *γ* and *β* secondary processes of PAzMa samples, is shown in Figure 12. It can be seen that both secondary relaxations are thermally activated processes and they exhibit Arrhenius behaviour. The activation energies (*E_a_*) associated with the *γ* and *β* processes for both PAzMa samples, obtained from the slopes of the Arrhenius plots are 33.0 ± 0.3 kJ·mol^−1^ (PAzMa1) and 35.2 ± 0.4 kJ·mol^−1^ (PAzMa2) for the high frequency process and 57.4 ± 0.6 kJ·mol^−1^ (PAzMa1) and 55.8 ± 1.0 kJ·mol^−1^ (PAzMa2) for the low frequency process. The fact that the *E_ai_* for both samples and both processes are similar suggests that these relaxations have the same side chain motions origin. The *E_aγ_* obtained for PAzMa1 sample is close to that corresponding to CEOEMA [9] (see Table 2), being slightly higher for PAzMa2 sample. On the other hand, the *E_aβ_* obtained for both PAzMa samples is similar but higher than that obtained for non-crosslinked poly(2-ethoxyethyl methacrylate), PEOEMA [9]. In this case, is not possible to compare with the crosslinked poly(2-ethoxyethyl methacrylate), CEOEMA since as was mentioned early, this process is suppressed by slightly crosslinking the PEOEMA chains [9].

**Figure 13:**
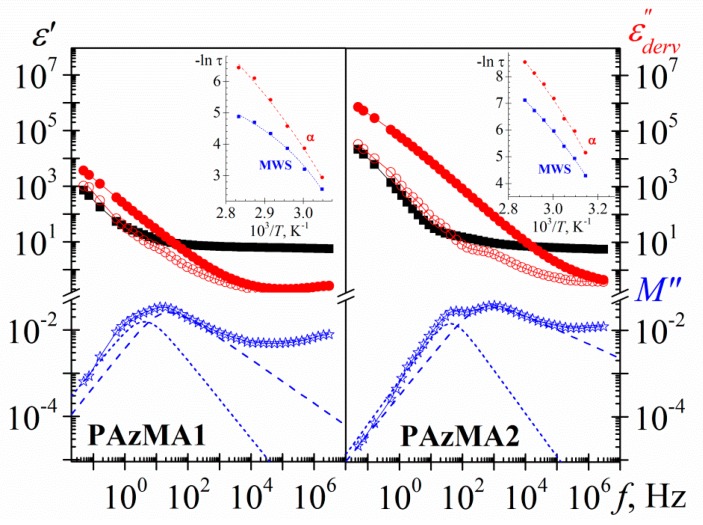
Frequency dependence of the dielectric constant (square), experimental loss permittivity (full circle), derivative loss permittivity (open circle) and derivative loss modulus (star) spectra of the PAzMA samples at 60 °C. Short dashed lines correspond to the MWS process and dashed lines to the *α* relaxation process. Inset: Temperature dependence of the relaxation time in the Arrhenius coordinates.

Figure 13 shows, for both PAzMA samples, the frequency dependence of the experimental complex dielectric permittivity (*ε’*, *ε”*) and the derivate loss permittivity and loss modulus (*ε”_der_*, *M”_der_*) at 60 °C. Observing the process associated with glass transition is possible only after removing the dc-conductivity contribution from the loss signal. As the display and definition of both processes is sharper in the loss modulus spectra, we have used the dielectric loss modulus derivate function in order to characterize them. Thus, the evaluation of the temperature dependence of the relaxation time of both, *α* relaxation and MWS processes, we have carried out by fitting the *M”_der_* spectra to the HN empirical model [7,30,31]. This analysis that was carried out only for those peaks in the dielectric spectra for which the peak maximum was clearly discernible. As follows from the insert in Figure 13, the *α* and MWS processes cannot be adequately described with the Arrhenius equation. As usual, the temperature dependence of the relaxation times for the *α*-relaxation is parameterized by means of the Vogel-Fulcher-Tammann-Hesse (VFTH) equation [32,33,34] The VFTH fitting parameters obtained for both PAzMA samples are summarized in Table 2. The value of the glass transition, evaluated from the VFTH fit parameters for *τ* = 100 s, are 52.7 °C (PAzMA1) and 37.6 °C (PAzMA2) [35]. These values are lower than those obtained by DSC analysis. The discrepancy between the values obtained by means both techniques can be understood considering the different probes measured by both methods. This tendency has been found by other authors and is directly related to the fact that whereas DRS sense fluctuations of dipoles DSC sense enthalpy or entropy fluctuations [36]. In addition to the foregoing, the differences found may be associated with the high heating scan rate used in DSC as well as to the uncertainty associated with the fitting procedure of the derived spectrum.

As in other heterogeneous systems [7,9,37], interfacial polarization arising from the build-up of charges at the nanodomains interfaces may be responsible for the symmetric relaxation (MWS process) observed in the derivate spectra. The deviation of the linearity observed in the Arrhenius plot of the MWS relaxation process, shown in insets Figure 13, suggests that the absorption follows non-Arrhenius dependence. We have used the VFTH equation in order to characterize this process. The values obtained in the fit are summarized in Table 2.

**Figure 14:**
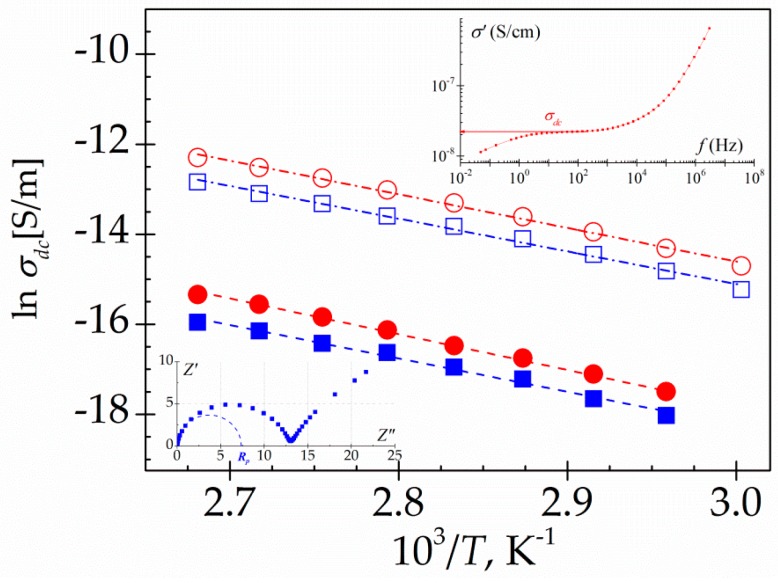
Temperature dependence of the dc conductivity (S/m) for PAzMa1 (full symbols) and PAzMa2 (open symbols) evaluated from the plateau at low temperatures of *σ’* versus *f* (circles) and from the Cole-Cole impedance plots (squares).

In order to analyse the charges transport process, which dominates the frequency dependence of the dielectric loss spectra in the low frequency zone, different procedures can be employed. One of them involves the analysis of the impedance data, another includes the analysis of the frequency dependence of the real part of the complex conductivity. Cole-Cole impedance plots are deformed arcs roughly described by [38]

(2) $$Z^{*}\left(\omega \right)=\frac{R_{p}}{{\left[1+{\left(i\omega \tau \right)}^{a}\right]}^{b}}\;$$

where *τ* is a mean-relaxation time, *R_p_* is the polarization resistance and, *a* and *b* are the shape parameters lie in the range 0 <*a*, *b*< 1. The arcs intersect the abscissa axis at the extreme frequencies in such a way that *Z’*(∞) = 0 and *Z’*(0) = *R_p_*. From *R_p_* we can estimate *σ_dc_* by using the relationship: $\sigma_{dc}=L/A\cdot R_{p}$, where *A* and *L* are the section and the thickness of the sample sandwiched between the electrodes, respectively (see inset Figure 14). The *σ_dc_* values can be also evaluated from the plateau at low frequencies of the *σ’* vs *f* plots [39,40] (see inset Figure 14). The values estimated by the two procedures are in reasonable good agreement as in shown in Figure 14. The conductivity of PAzMa2 is more of two decades higher than that of PAzMa1. This result indicates that the higher segmental motion of PAzMA2 favours the charge transport responsible of the conductivity process. In this sense, it has been proposed that the presence of intermolecular hydrogen bonds can give rise to a proton conduction process by an oscillation or interchange of the hydrogen provided by the amine group around the potential minimum of the neighbouring oxygen and nitrogen, respectively [6]. Some ab initio MD calculations carried out in several systems with possibility for both intra- and inter-chain transfers dynamical proton motion via the rearrangement of H-bonding network, emphasize the important role of this type of process in the conductivity values [41]. So, according to our results, the proton exchange path could be made easier with the addition of a new –CH_2_CH_2_O– unit in the side chain. For both samples, the conductivity process exhibits Arrhenius behaviour. As is showed in Table 2, the activation energy, obtained from the slopes of the Arrhenius plots, is similar for both samples (63.3 ± 1.8 kJ·mol^−1^ for PAzMa1 and 61.3 ± 1.6 kJ·mol^−1^ for PAzMa2), being lower than that of CEOEMA (93.9 ± 1.2 kJ·mol^−1^) [9].

## 4. Conclusions

Two PAzMa crosslinked samples with different length of oxyethylene pendant chain [–CONH–CH_2_CH_2_–(O–CH_2_CH_2_)_n_N_3_], n = 1 (poly(*N*-(2-(2-azidoethoxy)ethyl)methacrylamide), PAzMa1) or n = 2 (poly(*N*-2-(2-(2-azidoethoxy)ethoxy)ethyl)methacrylamide), PAzMa2, has been synthesized and analysed. The Young’s modulus, tensile strength and deformation depend on the length of the aliphatic side chain in such a way that the longer the lateral chain was, the lower Young’s modulus and tensile strength were and the higher the deformation at break were observed. Moreover, no significant changes in thermal stability were detected.

The DRS spectra of both samples exhibit two well-developed secondary absorptions labelled as *γ* and *β* relaxations in the glassy state. The oxyethylene pendant chain length does not have a significant effect in these secondary relaxation processes. According to the activation energies obtained in our analysis, the origin of these processes is probably similar to those of crosslinked poly(2-ethoxyethyl methacrylate). So, the *γ*-relaxation is, probably, associated with motions of the alcohol residue, whereas the *β*-relaxation is probably related to the local motions of the polymer backbone. However, a significant change in the strength of these processes is produced by the incorporation of a terminal azide group and the change of the –COO– group by the –CONH–group into the side chain. This change is attributed mainly to the higher dipole moment of the –CONH–group from the corresponding to the –COO– group.

The *α*-relaxation, associated with the glass transition has been characterized from the loss modulus derivate constant due to it is not visible in the experimental spectrum, which is dominated by the strong conductive processes at high temperatures and lower frequencies. The use of the derivative method based on the Kramer-Kroning relation, it has made possible the characterization of the glass transition process by fitting the loss derivate module to the HN model. The glass transition temperatures assessed by DRS using the VTF fit parameters and assuming that *τ* = 100 s is in reasonable agreement with the corresponding values evaluated from the DSC measurements. By increasing the length of the side chain a reduction in the glass transition temperature is observed. This result indicates a higher segmental motion in PAzMA2.

On the other hand, the conductivity increases for more than two decades by increasing the oligooxyethylene pendant chain length but the activation energy of the conductivity process remains practically unchanged, pointed to a similar origin for both. As the crosslinked content is the same in both samples, the results indicate that the increase in conductivity is associated directly with the greater segmental motion of molecular chains when longer the aliphatic side chain of the polymers is. Our results point than both, intra- and inter-chain transfers dynamical proton motion, via the rearrangement of H-bonding network, result easier with the addition of a new –CH_2_CH_2_O– unit in the side chain. Thereby, broadband dielectric spectroscopy is a very sensitive and selective tool to investigate the dynamics of these functional azide organic systems.

## Figures and Tables

**Table 1:** Thermal and mechanical properties of films PAzMa1 and PAzMa2. T5 and T10 are temperatures at which 5 and 10% weight loss are observed, respectively.

	Thermal Properties			Mechanical Properties		
Sample	T5 (°C)	T10 (°C)	Char Yield (%)	Young’s Modulus (MPa)	Tensile Strength (MPa)	Deformation at Break (%)
PAzMa1	198	222	7	778	25	4
PAzMa2	199	224	7	246	12	14

**Table 2:** Arrhenius ($\tau =\tau_{0}\cdot e^{-E_{a}/RT}$) and Vogel-Fulcher-Tammann-Hesse ($\tau =\tau_{0}\cdot e^{M/\left(T-T_{v}\right)}$) equations fit parameters for the dipolar and conductive analysed processes.

		PAzMa1	PAzMa2	PEOEMA *	CEOEMA *
***γ*-process**	*E_a_*, kJ·mol^−1^	33.0 ± 0.3	35.2 ± 0.4	29.9 ± 0.4	30.1 ± 0.4
	*τ*_0_, s	10^−14.3 ± 0.1^	10^−14.8 ± 0.1^	10^−14.0^	10^−14.0^
***β*-process**	*E_a_*, kJ·mol^−^^1^	56.1 ± 0.5	55.4 ± 1.2	41.6 ± 0.3	-
	*τ*_0_, s	10^−17.0 ± 0.1^	10^−16.4 ± 0.3^	10^−13.9^	-
***α*-process**	*τ*_0_, s	10^−7.8 ± 0.2^	10^−7.5 ± 2.1^	10^−11.0 ± 0.6^	10^−9.7 ± 0.1^
	*M*, *K*	992.9 ± 22.8	945.6 ± 53.1	1210.5 ± 69.3	1214.7 ± 18.2
	*T_v_*, *K*	255.7 ± 13.0	240.6 ± 20.1	220.1 ± 3.4	233.6 ± 1.0
**MWS-process**	*τ*_0_, s	10^−6.4 ± 0.2^	10^−6.3 ± 0.8^	-	-
	*M*, *K*	653.0 ± 17.7	621.5 ± 28.9	-	-
	*T_v_*, *K*	263.1 ± 2.1	252.5 ± 18.8	-	-
***σ*-process**	*E_a_^σ^*, kJ·mol^−^^1^	63.3 ± 1.8	61.3 ± 1.6	122.9 ± 0.8	93.9 ± 1.2
	*τ*_0_, s	10^−2.1 ± 0.3^	10^−3.2 ± 0.2^		

* Taking from ref [9].
